# Electric field control of magnetization direction across the antiferromagnetic to ferromagnetic transition

**DOI:** 10.1038/s41598-017-05611-7

**Published:** 2017-07-14

**Authors:** Guohui Zheng, San-Huang Ke, Maosheng Miao, Jinwoong Kim, R. Ramesh, Nicholas Kioussis

**Affiliations:** 1 Department of Physics and Astronomy, California State University Northridge, Northridge, California 91330 USA; 2 MOE Key Laboratory of Microstructured Materials, School of Physics Science and Engineering, Tongji University, 1239 Siping Road, Shanghai, 200092 P. R. China; 3 Materials Sciences Division, Lawrence Berkeley National Laboratory, Berkeley, CA 94720 USA

## Abstract

Electric-field-induced magnetic switching can lead to a new paradigm of ultra-low power nonvolatile magnetoelectric random access memory (MeRAM). To date the realization of MeRAM relies primarily on ferromagnetic (FM) based heterostructures which exhibit low voltage-controlled magnetic anisotropy (VCMA) efficiency. On the other hand, manipulation of magnetism in antiferromagnetic (AFM) based nanojunctions by purely electric field means (rather than E-field induced strain) remains unexplored thus far. *Ab initio* electronic structure calculations reveal that the VCMA of ultrathin FeRh/MgO bilayers exhibits distinct linear or nonlinear behavior across the AFM to FM metamagnetic transition depending on the Fe- or Rh-interface termination. We predict that the AFM Fe-terminated phase undergoes an E-field magnetization switching with large VCMA efficiency and a *spin reorientation* across the metamagnetic transition. In sharp contrast, while the Rh-terminated interface exhibits large out-of-plane (in-plane) MA in the FM (AFM) phase, its magnetization is more rigid to external E-field. These findings demonstrate that manipulation of the AFM Néel-order magnetization direction via purely E-field means can pave the way toward ultra-low energy *AFM-based* MeRAM devices.

## Introduction

Spintronics offers a promising solution^1, 2^ to the major challenging issues related to the scaling of Si-based complementary metal-oxide-semiconductor (CMOS) technology because of the advantages of combing the spin and charge degrees of freedom and its ability to manipulate magnetic states in low-power-consumption ways^3, 4^. The discovery of giant magnetoresistance (GMR)^5, 6^ and tunnel magnetoresistance (TMR)^7, 8^ allowed electrical readout of the relative orientation of magnetic moments in spin valves or magnetic tunnel devices consisting of two ferromagnetic metallic layers separated by a very thin non-magnetic metal or insulator spacer. More recently, it has been demonstrated^9, 10^ that the magnetic state of a nanoscale MTJ can be switched by a spin-polarized tunnel current via the so-called spin-transfer torque (STT) involving the transfer of spin angular momentum between the noncollinear magnetization of the ferromagnetic layers^11, 12^. Even though the STT offers a promising new mechanism for the write operation of nanomagnetic memory element it inevitably involves Joule heating and hence has high power consumption.

Electric field induced switching of magnetism, as opposed to current-driven spin transfer torque magnetization switching, can lead to a new paradigm enabling ultra-low power, highly scalable, and nonvolatile magnetoelectric random access memory (MeRAM)^13–15^. To date the realization of MeRAM relies primarily on ferromagnetic (FM) based heterostructures consisting of heavy metal (HM/)FM/insulator(HM = Ta, Hf, Mo) nanojunctions^13, 14, 16–18^. The major bottleneck in optimizing the performance of MeRAM devices is the low voltage-controlled magnetic anisotropy (VCMA) efficiency, *β*, (change of interfacial MA energy per unit electric field) which is typically <80 fJ/(Vm), leading in turn to high switching energy per bit (~100 fJ) and high write voltage (>2 V)^19^. *Ab initio* calculations of the efficiency of the linear VCMA range from about +130 to +70 fJ/(Vm) for Fe/MgO^20^ to Au/Fe/MgO^21^ nanojunctions. Recently, we have predicted that epitaxial strain in HM/FM/insulator heterostructures gives rise to giant VCMA efficiency^22, 23^.

On the other hand, antiferromagnetic (AFM) materials, with staggered magnetic order accompanied by a zero net magnetic moment, have been revisited as potential candidates for active elements in spintronic devices^24^. In contrast to their FM counterparts, AFM systems have weak sensitivity to magnetic field perturbations, produce no perturbing stray fields, and can offer ultra-fast writing schemes^25^. The AFM Néel-order spin direction can be controlled indirectly by a magnetic field via an attached exchange-coupled FM^26^ or by techniques analogous to heat-assisted magnetic recording^27^ or by a lateral electric current via Néel order spin-orbit torque fields that alternate in sign between the two sublattices^28^. However the energy efficiency of these methods for controlling the AFM spin direction is limited.

**Figure 1:**
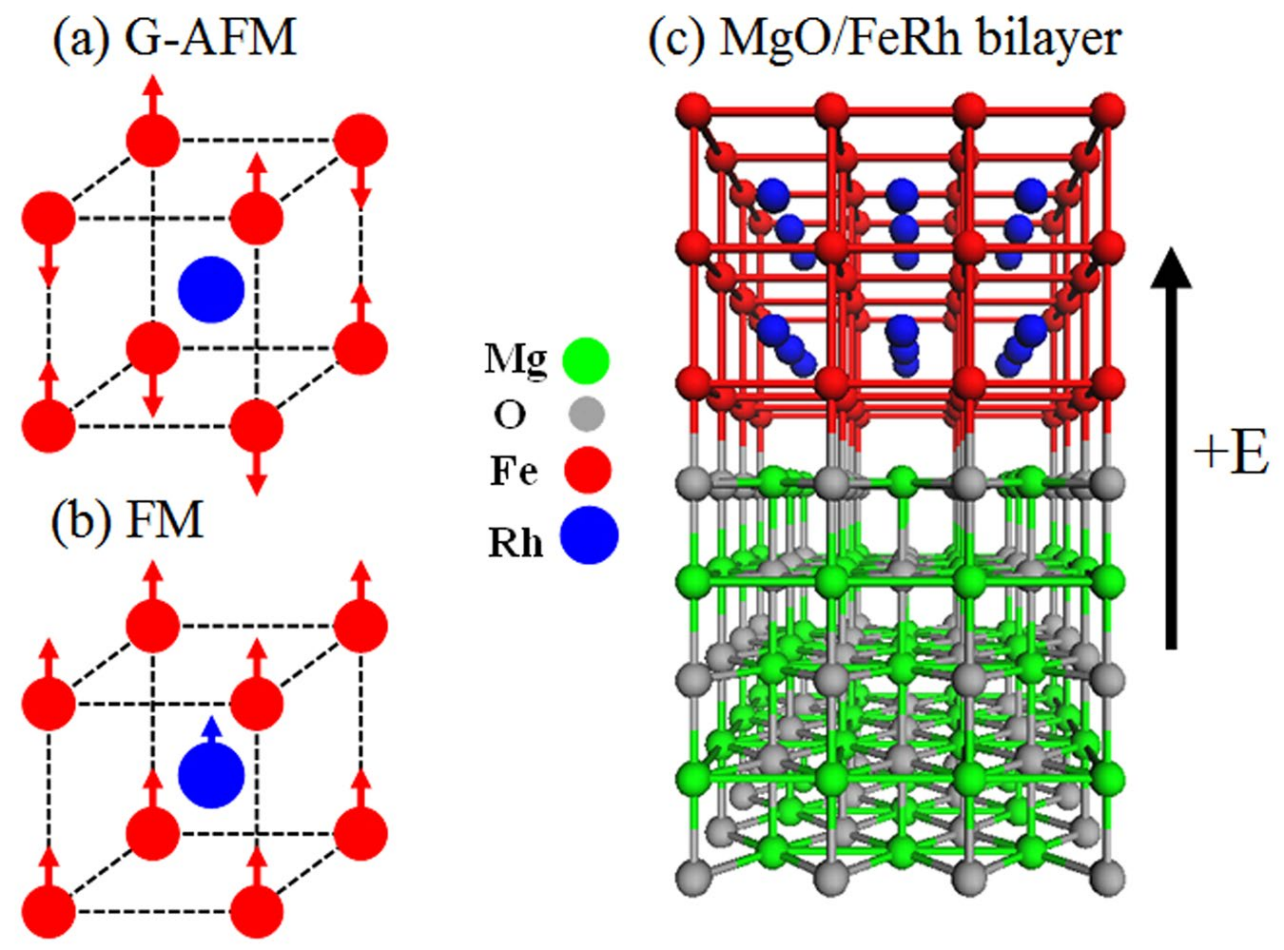
Magnetic structures of the (**a**) G-AFM and (**b**) FM phases of bulk FeRh. In the G-AFM structure the Fe atoms have staggered local moments of ~±3.2 *μ*_*B*_ which align antiferromagnetically between nearest-neighbor Fe atoms while the Rh has zero moment. In the FM phase the Rh moment is ~1 *μ*_*B*_. (**c**) The Fe-terminated interface and surface of the FeRh/MgO bilayer structure. Also we show the positive direction of the external electric field.

Among AFM metals the near equiatomic chemically ordered bcc-B2 (CsCl-type) bulk FeRh alloys are prototype systems that continue to attract intense interest due to a wide range of intriguing magnetic properties and their potential applications in thermally assisted magnetic recording media^29^, magnetic cooling^30^, ultrafast (ps) switching^31^, and room-temperature antiferromagnetic memory resistor^27^. FeRh undergoes an unusual first-order phase transition from AFM to FM order at ~350 K which is accompanied by volume expansion of ~1% indicating coupling between the magnetic and structural degrees of freedom^32, 33^. The left panels in Fig. 1 show the G-type AFM and FM structures, respectively. The G-AFM ordering (Fig. 1(a)) can be described as alternating (111) FM planes which are antiferromagnetically coupled along the sheet normal direction, where the Fe has a local moment of ~+3.2 *μ*_*B*_ and the Rh has a negligible moment. In contrast, in the high-temperature FM phase (Fig. 1(b)) the iron and rhodium local moments are ~3 *μ*_*B*_ and ~1*μ*_*B*_, respectively^32, 33^ and the nonzero Rh local moment is induced by the non-vanishing net exchange field from the nearest-neighbor Fe atoms. The underlying origin for this transformation is controversial and remains unresolved. Proposed mechanisms include changes in the electronic entropy^34^, spin-wave excitations^35^, instability of the Rh magnetic moment^36^, and magnetic excitations^37^.

In recent years particular interest has grown on controlling the magnetism in FeRh thin films grown epitaxially on MgO^38^, BaTiO_3_^39, 40^ and piezoelectric^41^ substrates. These experiments demonstrated spin reorientation across the AFM-FM phase transition^38^ and isothermal electric field control of the magnetic phase transition driven via tetragonal piezoelectric strain^39–41^. Nevertheless, manipulation of the AFM magnetization direction of ultrathin (~1 nm) FeRh/insulator bilayers in the AFM or FM phase by purely electric field means (rather than E-field induced strain), is of fundamental importance for the operation of the next-generation ultra-low power MeRAM and has not been investigated thus far.

In this work, we employ *ab initio* electronic structure calculations to study systematically the effect of electric field, strain and surface termination on the magnetic anisotropy of ultrathin FeRh/MgO bilayers across the metamagnetic transition. We find that the VCMA behavior for the Fe-terminated interface is nonlinear for both the AFM and FM phases with large VCMA efficiency including an E-field magnetization reversal in the AFM phase. On the other hand, the VCMA behavior of the Rh-terminated interface is linear for both the FM and AFM phases with smaller VCMA efficiency indicating the Rh magnetic moments are more rigid to external E-field. Both Fe- and Rh-terminated interfaces show a spin reorientation across the metamagnetic transition in agreement with experiment.

## Results and Discussion

### Effect of strain on stability of magnetic phases

For the Fe-terminated surface we find that the G-AFM is the most stable phase for both the free standing 5-ML FeRh and FeRh/MgO thin films regardless of the strain on FeRh (−0.5% < *η*_*FeRh*_ < 0.5%) with ${\rm{\Delta }}E={E}_{FM}-{E}_{G-AFM}$ of $\simeq$ 23 meV/Fe and 20 meV/Fe, respectively. For comparison, recent *ab initio* calculations of the energy landscape of the G-AFM and FM structures of bulk FeRh as a function of volume and tetragonal distortion show that the energy difference between the *cubic* G-AFM and FM structures is 64.5 meV per formula unit^42^. Interestingly, experiments in FeRh films of thickness ~10 nm report^43^ that the transition temperature is reduced to ~300 K from the bulk value of about 380 K, indicating that the FM state is becoming more stable than the G-AFM state with decreasing film thickness, consistent with our results. On the other hand, for the Rh-terminated surface we find that the FM structure is the ground state, where |Δ*E*| is $\simeq$ 134 meV/Fe and 97 meV/Fe for the FeRh and FeRh/MgO thin films, respectively. These results indicate that the MgO substrate decreases the energy difference between the G-AFM and FM phases. Our *ab initio* results are consistent with recent experiments^44^ which find evidence that the Rh-terminated surface of FeRh is FM at room temperature while the bulk is in the AFM phase. Furthermore, the results for free-standing FeRh films are consistent with previous DFT calculations^45, 46^.

### Strain-dependent zero field MA

**Figure 2:**
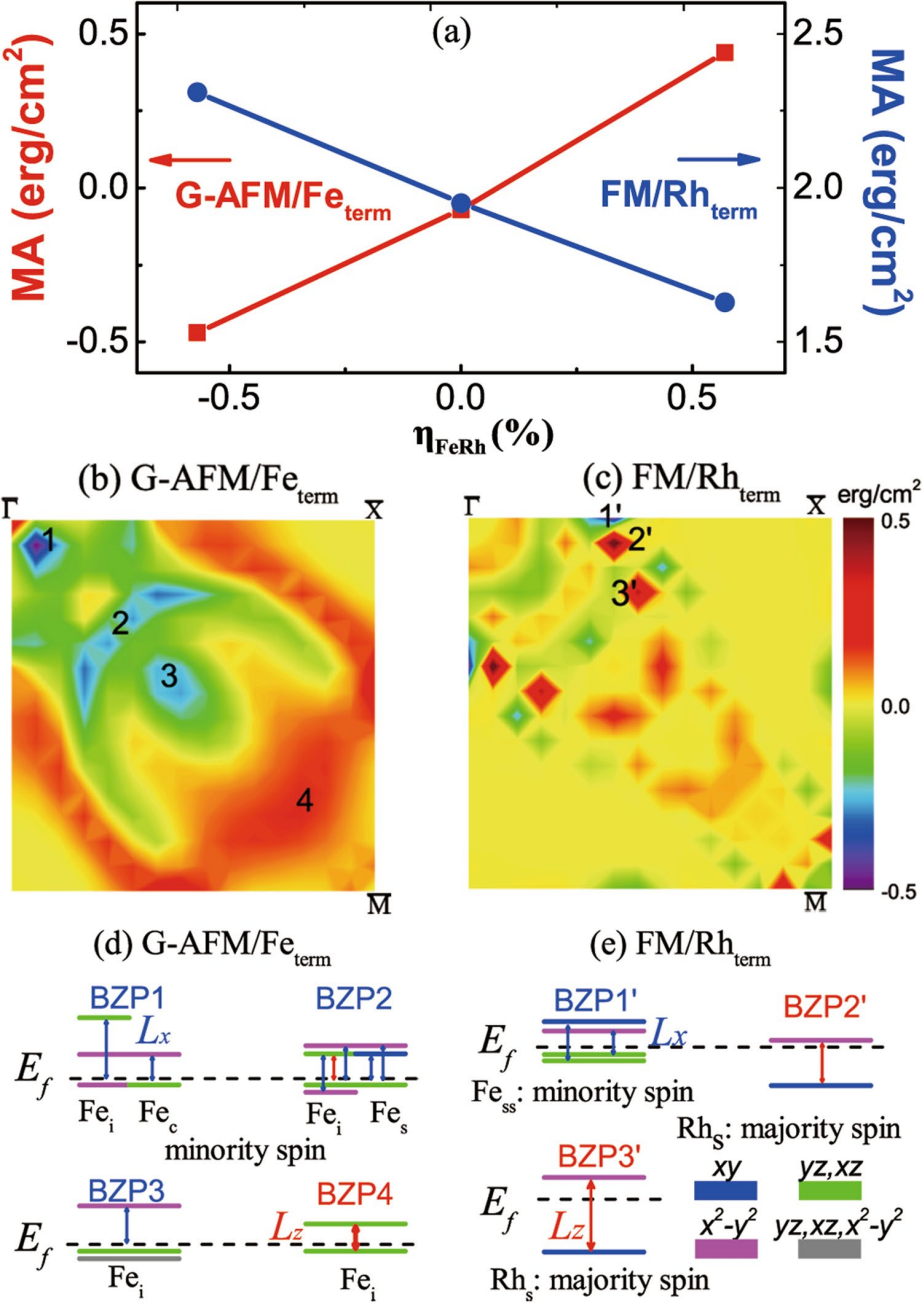
(**a**) Strain dependence of zero-field MA of the G-AFM Fe-terminated (left-hand coordinate, solid square) and the FM Rh-terminated surfaces/interfaces (right-hand coordinate, solid circle) for the FeRh/MgO bilayer. **k**-resolved MA (in *erg*/*cm* ^2^) in the quarter of the 2D BZ for the (**b**) G-AFM Fe-terminated and (**c**) FM Rh-terminated phases for *η*_*FeRh*_ = −0.57%. Numerals refer to special BZPn (*n* = 1–4,1′–3′) *k*-points where the MA values are extrema. (**d**) Fe-terminated G-AFM phase under *η*_*FeRh*_ = −0.57%: Low energies of the minority-spin Fe-*d*-derived orbitals relative to the Fermi energy (dashed horizontal line) at various BZPn for the interfacial, Fe_*i*_, surface Fe_*s*_, and central Fe_*c*_ atoms. (**e**) Rh-terminated FM phase under *η*_*FeRh*_ = −0.57%: Low energies of the minority-spin *d*-derived orbitals of subsurface Fe_*ss*_ atom at BZP1′ and of the majority-spin *d*-derived orbitals of the surface Rh_*s*_ atom at BZP2′ and BZP3′, respectively. Red and blue vertical lines denote non-vanishing SOC matrix elements of the out-of (${\hat{L}}_{z}$) and in-plane (${\hat{L}}_{x}$) orbital angular momentum operator, respectively.

In Fig. 2(a) we show the variation of zero-field MA with strain, *η*_*FeRh*_, for the stable G-AFM Fe-terminated and the FM Rh-terminated FeRh/MgO surfaces/interfaces, respectively. We find that the Fe-terminated G-AFM FeRh/MgO bilayer undergoes a transition from an in- to out-of-plane magnetization with increasing biaxial strain while the Rh-terminated FM bilayer which has large spin-orbit coupling (SOC) exhibits large out-of-plane orientation (note the different scale in the left- and right-handed coordinate). The strain-dependent MA can be expressed as^22^, $MA=2{B}_{1}t{\eta }_{FeRh}+{K}_{2}^{i}$, where *t* is the FeRh film thickness, ${K}_{2}^{i}$ is the effective interfacial uniaxial and the magnetoelastic coefficient, ${B}_{1}={B}_{1}^{v}+{B}_{1}^{i}/t$, magnetocrystalline anisotropy, is the sum of volume (${{\rm{B}}}_{1}^{v}$) and interface (${{\rm{B}}}_{1}^{i}$) contributions. Fitting the calculated MA to the above expression we find that ${K}_{2}^{i}=$ −0.03 erg/cm^2^ and 1.62 erg/cm^2^ for the G-AFM/Fe-terminated and FM/Rh-terminated surface/interface. The MA for the FM Fe-terminated and G-AFM Rh-terminated interfaces under zero strain is 0.55 erg/cm^2^ and −1.5 erg/cm^2^, respectively, demonstrating that thin FeRh/MgO bilayers undergo a spin reorientation across the AFM-FM metamagnetic transition in agreement with recent experiments^38^.

In order to elucidate the underlying origin of the dependence of the MA on surface/inteface termination we show in Fig. 2(b) and (c) the **k**-resolved $MA(k)\approx {\sum }_{n\in occ}[\varepsilon {(n,k)}^{\mathrm{[100]}}-\varepsilon {(n,k)}^{\mathrm{[001]}}]$ ^47^ in the 2D Brillouin zone (BZ) for the G-AFM Fe- and FM Rh-terminated interfaces, respectively, for *η*_*FeRh*_ = −0.57%. Here, $\varepsilon {(n,k)}^{\mathrm{[100]([001])}}$ are the eigenvalues of the Hamiltonian for magnetization along the [100] ([001]) direction. Overall, we find that the MA values determined from total energy calculations agree well with those determined from the force theorem^47^ where $MA={\sum }_{{\bf{k}}}MA({\bf{k}})$. Even though the MA(*k*) exhibits *hot spots* (special **k** points in the 2D BZ) for both terminations where the MA has positive or negative contributions, its texture is very different due the distinct energy- and **k**-resolved distribution of the minority- and majority spin bands of the surface and interfacial Fe or Rh atoms.

Within second-order perturbation theory the MA can be expressed as^48^:

1 $$\begin{array}{lll}MA & \propto  & {\xi }^{2}\,\sum _{o,u}\,\frac{{|\langle {{\rm{\Psi }}}_{o}^{\downarrow }|{\hat{L}}_{z}|{{\rm{\Psi }}}_{u}^{\downarrow }\rangle |}^{2}-{|\langle {{\rm{\Psi }}}_{o}^{\downarrow }|{\hat{L}}_{x}|{{\rm{\Psi }}}_{u}^{\downarrow }\rangle |}^{2}}{{E}_{u}^{\downarrow }-{E}_{o}^{\downarrow }}\\  &  & +\mathrm{majority} \mbox{-} \mathrm{spin}\,{\rm{term}}+\mathrm{mix} \mbox{-} \mathrm{spin}\,{\rm{term}},\end{array}$$

where ${{\rm{\Psi }}}_{o}^{\downarrow }$(${E}_{o}^{\downarrow }$) and ${{\rm{\Psi }}}_{u}^{\downarrow }$(${E}_{u}^{\downarrow }$) are the one-electron occupied and unoccupied minority-spin states (energies) of band index n and wave vector **k** (omitted for simplicity), *ξ* is the SOC constant, and ${\hat{L}}_{x(z)}$ is the *x*(z) component of the orbital angular momentum operator. This expression allows to understand the underlying origin of the effect of strain or E-field on the MA.

Figure 2(d) and (e) show the pertinent low-energy levels of the interfacial, surface, and central Fe or Rh atoms relative to the Fermi energy at specific BZ **k**-points (BZPn) for the Fe- and Rh-terminated interface, respectively, where the MA assumes its extremum values. For the Fe-terminated surface/interface [2(d)], the energy- and **k**-resolved analyses of the spin bands suggest that only the minority spin of Fe atoms contributes significantly to the total MA. The negative MA at BZP1 is due to the SOC of the minority spin interfacial Fe-derived occupied ${d}_{{x}^{2}-{y}^{2}}$ states with the unoccupied minority-spin *d*_*yz*(*xz*)_ states and of the minority-spin central Fe-derived *d*_*yz*(*xz*)_ states with the unoccupied minority-spin ${d}_{{x}^{2}-{y}^{2}}$ states through the ${\hat{L}}_{x}$ operator, respectively. Similarly, the negative MA at BZP2 and BZP3 is due to the large SOC of the interfacial and central Fe-derived minority-spin $\langle {x}^{2}-{y}^{2}|{\hat{L}}_{x}|xz(yz)\rangle$ and $\langle xz(yz)|{\hat{L}}_{x}|{x}^{2}-{y}^{2}\rangle$, respectively. The positive $\langle xz|{\hat{L}}_{z}|yz\rangle$ contribution of *Fe*_*i*_ at BZP2 is crucial on the variation of the MA under negative E-field to be discussed below. On the other hand, the SOC $\langle xz|{\hat{L}}_{z}|yz\rangle$ at BZP4 of Fe_*i*_ yields a positive contribution to the MA.

For the Rh-terminated surface/interface [2(e)], similar analyses show that the minority spin of subsurface Fe atoms and majority spin of the surface Rh atoms also contribute to the MA at its hot spots. The negative MA at BZP1′ arises from the SOC of the minority-spin subsurface Fe-derived $\langle xz(yz)|{\hat{L}}_{x}|{x}^{2}-{y}^{2}\rangle$ and $\langle xz(yz)|{\hat{L}}_{x}|xy\rangle$. On the other hand, the positive MA at BZP2′ and BZP3′ is due the SOC of the majority-spin surface Rh-derived occupied *d*_*xy*_ states with the unoccupied ${d}_{{x}^{2}-{y}^{2}}$ states through the ${\hat{L}}_{z}$ operator.

### Effect of interface-termination on VCMA across AFM-FM transition

**Figure 3:**
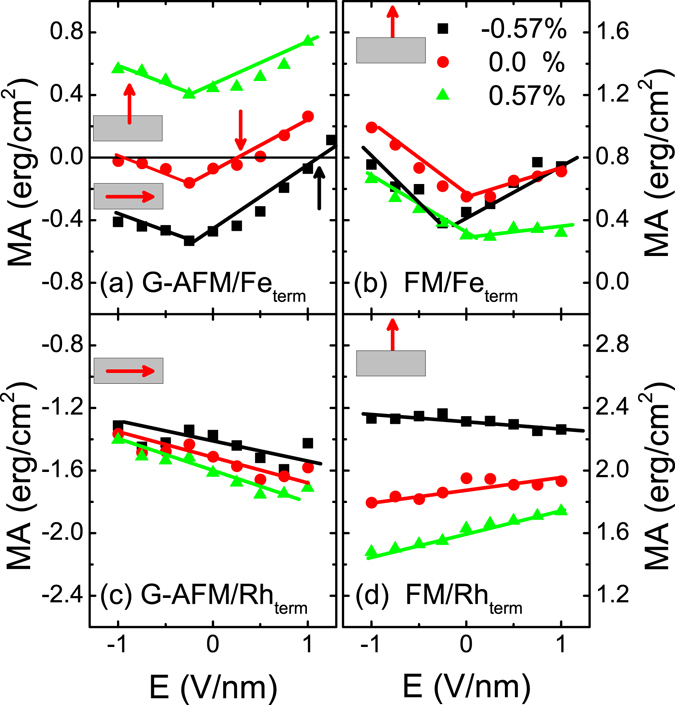
MA versus E-field in MgO for the Fe-terminated surface/interface film in the (**a**) G-AFM and (**b**) FM phases; and for the Rh-terminated surface/interface in the (**c**) G-AFM and (**d**) FM phases for different strain values. Note the different scale in left- and right-hand coordinate.

In the low-bias regime the VCMA is proportional to the E-field in the insulator, $VCMA=\beta {E}_{MgO}=\beta {E}_{ext}/\varepsilon$, where *β* is the VCMA coefficient, *ε* = 10 is the dielectric constant of the MgO in the range of strain (~±0.57%)^22, 23^, and *E*_*ext*_ is the external E-field^22^. The variation of MA as a function of the E-field in MgO for the Fe-terminated FeRh/MgO bilayer in the G-AFM (ground state) and the FM phase for different strain is shown in Fig. 3(a) and (b), respectively; and that for the Rh-terminated bilayer in the G-AFM and FM (ground state) is shown in Fig. 3(c) and (d), respectively. The calculations show that the interface/surface termination and the strain have a dramatic effect on the VCMA behavior.

### Fe-termination

For the G-AFM/Fe-terminated bilayer the results show that the VCMA has a robust asymmetric ∨-shape regardless of strain with *giant β* values of +360 (−180) fJ/(Vm) for positive (negative) E-field for *η*_*FeRh*_ = −0.57%; to +300 (−230) fJ/(Vm) for zero strain; and to +200 (−250) fJ/(Vm) for *η*_*FeRh*_ = +0.57%. Similar ∨-shape E-dependence of MA has been reported in FM-based trilayers^16, 22, 49^. More importantly, we predict an *E-field-driven switching* of the AFM Néel-order magnetization direction from in- to out-of-plane direction at about 1.1 (+0.28) V/nm for *η*_*FeRh*_ = −0.57% (0%), denoted by the vertical black (red) arrows (the breakdown electric field of MgO is about 1.2 V/nm^50^). In contrast to the recently demonstrated room-temperature AFM memory resistor^27^ which involves heating up FeRh above the AFM − FM transition temperature and subsequently field-cooling below the transition temperature the proposed low-power room-temperature E-field spin reorientation of AFM Fe-terminated FeRh films will improve the energy efficiency and read/write speed for the next generation of miniaturized memory and logic devices. We would like to emphasize that the predicted VCMA coefficient values are higher than (1) the critical value of ~200 fJ/(Vm) required to achieve a switching bit energy below 1fJ in the next-generation of MeRAMs^19^ and (2) by about one order of magnitude compared to those experimentally reported in FM-based heterostructures^14, 18^.

For the FM/Fe-terminated bilayer [Fig. 3(b)] the E-field behavior of the MA has also asymmetric ∨-shape regardless of strain with *giant β* values of +330 (−550) fJ/(Vm) for positive (negative) E-field for *η*_*FeRh*_ = −0.57%; to +160 (−440) fJ/(Vm) for zero strain; and to +70 (−420) fJ/(Vm) for *η*_*FeRh*_ = +0.57%. However, the FM/Fe terminated film exhibits a large perpendicular MA (PMA) regardless of strain for the entire range of E-field in sharp contrast with the G-AFM Fe-terminated interface. The results also demonstrate that there is a spin reorientation of the Fe-terminated FeRh film for a wide range of E-field and strain as the FeRh undergoes a transition from the G-AFM to the FM phase.

### Rh-termination

The variation of the MA of the G-AFM/Rh-terminated bilayer with E-field in Fig. 3(c) shows linear behavior in the entire strain range with *β* values of −130 fJ/(Vm) for *η*_*FeRh*_ = −0.57%; to −160 fJ/(Vm) for zero strain; and to −200 fJ/(Vm) for *η*_*FeRh*_ = +0.57%, respectively. Note that the MA is large (due to the large SOC of Rh) and negative indicating an in-plane magnetization orientation in the entire E-field range. For the FM/Rh-terminated film the linear E-field dependence of the MA, shown in Fig. 3(d), exhibits large PMA (1.4–2.2 erg/cm^2^) which depends sensitively on strain with *β* values of −50 fJ/(Vm) for *η*_*FeRh*_ = −0.57%; to 80 fJ/(Vm) for zero strain; and to +120 fJ/(Vm) for *η*_*FeRh*_ = +0.57%, respectively. Overall, while the large SOC of the Rh-terminated interface/surface enhances the zero-field MA (either in- or out-of-plane) the VCMA efficiency (*β*) is lower than that of the Fe termination indicating that the Rh moments are more rigid to external electric field.

**Figure 4:**
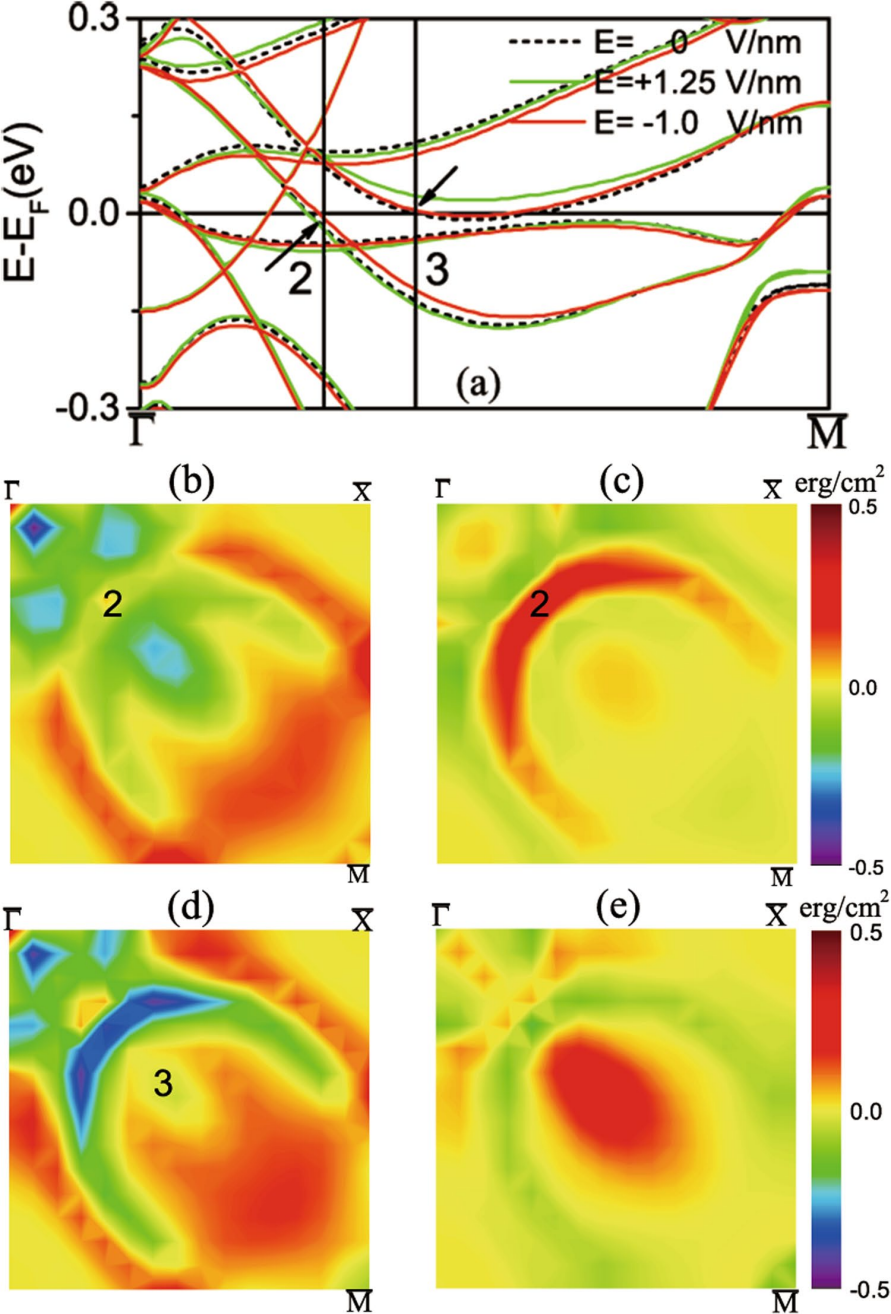
G-AFM/Fe-terminated FeRh/MgO bilayer under −0.57% strain: (**a**) Shift of minority-spin bands along the $\overline{{\rm{\Gamma }}{\rm{M}}}$ direction under zero (black curves), +1.25 V/nm (green curves) and −1 V/nm (red curves), respectively. (**b**) **k**-resolved MA in quarter of 2D BZ under −1 V/nm. (**c**) ΔMA(**k**) = MA(**k**, E) -MA(**k**, E = 0), under −1 V/nm. (**d**) **k**-resolved MA in quarter of 2D BZ under +1.25 V/nm. (**e**) ΔMA(**k**) = MA(**k**, E)-MA(**k**, E = 0), (in erg/cm^2^) under +1.25 V/nm.

Figure 4(a) shows the band structure of the G-AFM/Fe-terminated FeRh/MgO along the $\overline{\Gamma M}$ direction under zero (black curves), +1.25 V/nm (green curves), and −1 V/nm (red curves), respectively. The BZPn (n = 2–3) **k**-points in Fig. 4(a)–(e) are identical to those in Fig. 2(b) where the MA values assume extremum values. We find that the highest occupied minority-spin *d*(*xz*, *yz*)-derived bands of the interfacial Fe atom at BZP2 [Fig. 2(d)] shift upward closer to the Fermi energy under negative E-field. Furthermore, the highest occupied minority-spin *d*(*xz*, *yz*)-derived bands of Fe_*i*_ at BZP3 shift above the Fermi energy under positive E-field. These E-field induced changes of the band energies at various *hot* spots change in turn MA(**k**)(*E*) and hence the field-induced ΔMA(**k**) = MA(**k**, *E*) − MA(**k**, *E* = 0), which are shown in Fig. 4(b) and (c), respectively, for −1 V/nm, and in Fig. 4(d) and (e), respectively, for +1.25 V/nm. Thus, the negative E-field in Fig. 4(c) increases the positive $\langle xz|{\hat{L}}_{z}|yz\rangle$ contribution of *Fe*_*i*_ at BZP2 rendering ΔMA(BZP2)>0. Under positive field in Fig. 4(d) and (e) the occupied frontier *d*(*xz*, *yz*)-derived valence band at BZP3 in Fig. 4(a) shifts above the Fermi energy thus eliminating the SOC $\langle xz(yz)|{\hat{L}}_{x}|{x}^{2}-{y}^{2}\rangle$ under zero field. In addition, this shift induces a strong SOC between the occupied $d(xz,yz,{x}^{2}-{y}^{2})$- derived valence band [grey level in Fig. 2(d)] and the unoccupied *d*(*xz*, *yz*)-derived band (shifted above *E*_*F*_ by the positive field) through the out-of-plane orbital angular momentum, ${\hat{L}}_{z}$, rendering ΔMA(BZP2) > 0.

The underlying origin of the ∨-shape E-field behavior presumably arises from the fact that the interface bands and their E-field-induced shift depend on the magnetization direction due to the Rashba effect. The Rashba coupling, which is proportional to the *net* electric field, *E*_*z*_, at the interface, has contributions from both the internal and external fields^51^. The critical field where the magnetic anisotropy energy reaches its maximum or minimum value depends on the interplay between the two E-fields, where the internal E-field can be tuned via strain or interface termination. Interestingly, recent experiment has reported the effect of the internal electric field at ferromagnetic/insulator interface on the voltage-dependent tunneling anisotropic magnetoresistance^52^.

In summary, we predict a wide range of novel and interesting VCMA behavior of FeRh/MgO nanojunctions which depends sensitively on the magnetic phase and interface termination, including (i) an E-field switching of the AFM Néel-order magnetization direction with large VCMA efficiency, (ii) a spin re-orientation across the AFM-FM transition, and (iii) stronger (weaker) magnetization rigidity to external field for the Rh- (Fe-) terminated interface. These findings suggest the E-field tuning of the magnetic direction for ultrathin magnetic heterostructures that undergo AFM-FM metamagnetic phase transition.

## Methods

The *ab initio* calculations have been carried out within the framework of the projector augmented wave formalism^53^, as implemented in the Vienna *ab initio* simulation package (VASP)^54–56^. We employ the Perdew-Burke-Ernzerhof (PBE) exchange-correlation functional^57^. The energy cutoff of the plane-wave expansion of the basis functions was set to be 500 eV. We use a slab with odd-number of FeRh monolayers to ensure that the magnetic anisotropy of both the surface and interface layers arises from the same type of atoms. The slab supercell (Fig. 1(c)) for the FeRh/MgO (001) bilayer along [001] consists of five monolayers (ML) of FeRh with two Fe or Rh atoms per layer on top of five MLs of rock-salt MgO and a 12-Å thick vacuum region, where the <110> axis of FeRh is aligned with the <100> axis of MgO. At the FeRh/MgO interface, the O atoms are placed atop of the Fe or Rh atoms for the Fe- and Rh-terminated interfaces, respectively. The dipole layer method^58, 59^ implemented in VASP is used to introduce the electric field and to correct the dipole moment present in FeRh slab due to asymmetric slab geometry. The direction of positive field is defined as pointing from MgO to FeRh (Fig. 1(c)).

For each in-plane lattice constant, the magnetic and electronic degrees of freedom and the atomic positions along [001] are relaxed *in the presence of the E-field* until the forces acting on the ions become less than 0.01 eV/Å. The calculated equilibrium lattice constant, *a*, for the G-AFM an FM FeRh phases are 2.995 Å and 3.012 Å, respectively, in good agreement with experiment^33^. The lattice constant mismatch between MgO (4.212 Å) and FeRh introduces a biaxial strain of about 0.5% to 1% for the FM and AFM FeRh phases, respectively, which can in turn play a crucial role on the VCMA of the bilayer. Employing a 31 × 31 × 1 k-point mesh the MA per interfacial area is determined from $MA=[{E}_{\mathrm{[100]}}-{E}_{\mathrm{[001]}}]/A$, where *E*_[100]_ and *E*_[001]_ are the total energies with magnetization along the [100] and[001] directions, respectively.
